# Supplementary material for: Predicting visual function by interpreting a neuronal wiring diagram
Source: Nature. 2024 Oct 2;634(8032):113–23. doi: 10.1038/s41586-024-07953-5 (PMC11446822; doi:10.1038/s41586-024-07953-5)
Supplement: Supplementary file 6 — CRF and ERF predictions for individual TmY4 and TmY9 cells. Analogous to Supplementary Data 3, but for TmY target types. Shown are the top four monosynaptic pathways, the strongest pathway passing through each of the top ten intermediary types (ranking from Extended Data Fig. 7), and the trisynaptic pathway Tm1–TmY–Dm3–TmY (see the section entitled Prediction of spatial normalization). [file 41586_2024_7953_MOESM6_ESM.zip › DataS4/TmY4/720575940643192224.pdf]

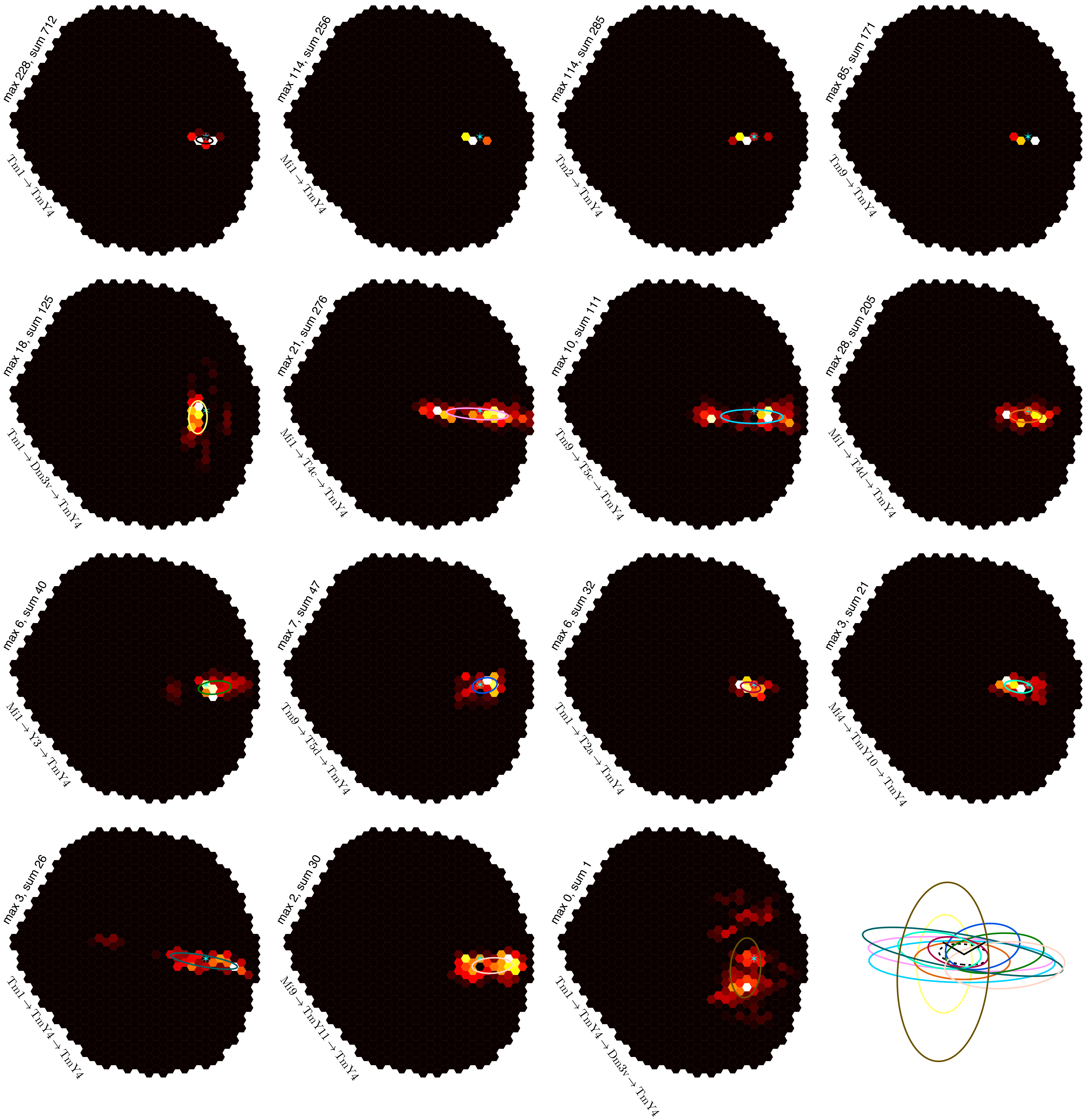
max 228, sum 712
Tm1 → TmY4
max 114, sum 256
Mi1 → TmY4
max 114, sum 285
Tm2 → TmY4
max 85, sum 171
Tm9 → TmY4
max 18, sum 125
Tm1 → Dm3v → TmY4
max 21, sum 276
Mi1 → T4c → TmY4
max 10, sum 111
Tm9 → T5c → TmY4
max 28, sum 205
Mi1 → T4d → TmY4
max 6, sum 40
Mi1 → Y3 → TmY4
max 7, sum 47
Tm9 → T5d → TmY4
max 6, sum 32
Tm1 → T2a → TmY4
max 3, sum 21
Mi4 → TmY10 → TmY4
max 3, sum 26
Tm1 → TmY4 → TmY4
max 2, sum 30
Mi9 → TmY11 → TmY4
max 0, sum 1
Tm1 → TmY4 → Dm3v → TmY4
